# Toward FAIR Representations of Microbial Interactions

**DOI:** 10.1128/msystems.00659-22

**Published:** 2022-08-25

**Authors:** Alan R. Pacheco, Charlie Pauvert, Dileep Kishore, Daniel Segrè

**Affiliations:** a Institute of Microbiology, ETH Zurich, Zurich, Switzerland; b Functional Microbiome Research Group, Institute of Medical Microbiology, University Hospital of RWTH, Aachen, Germany; c Bioinformatics Program and Biological Design Center, Boston University, Boston, Massachusetts, USA; d Department of Biology, Department of Biomedical Engineering, Department of Physics, Boston University, Boston Massachusetts, USA; Tufts University

**Keywords:** microbiome, microbial interactions, microbial ecology, data sharing, accessibility, reproducibility, FAIR, metadata, co-occurrence, microbial networks

## Abstract

Despite an ever-growing number of data sets that catalog and characterize interactions between microbes in different environments and conditions, many of these data are neither easily accessible nor intercompatible. These limitations present a major challenge to microbiome research by hindering the streamlined drawing of inferences across studies. Here, we propose guiding principles to make microbial interaction data more findable, accessible, interoperable, and reusable (FAIR). We outline specific use cases for interaction data that span the diverse space of microbiome research, and discuss the untapped potential for new insights that can be fulfilled through broader integration of microbial interaction data. These include, among others, the design of intercompatible synthetic communities for environmental, industrial, or medical applications, and the inference of novel interactions from disparate studies. Lastly, we envision potential trajectories for the deployment of FAIR microbial interaction data based on existing resources, reporting standards, and current momentum within the community.

The enormous progress in biotechnological and computational techniques over the last few decades has revolutionized our understanding of microbial communities. In particular, studies based on amplicon and metagenomic sequencing have further clarified the fact that microbiomes are not static entities, but dynamic ecosystems whose constituent members interact in myriad ways with each other and with their environments (1–6). These sequence-based approaches have catalyzed early efforts to map microbial interrelationships and represent an invaluable first step in identifying the organisms participating in these interactions. However, these approaches have largely been limited to inferring microbial associations (e.g., via co-occurrences) (7, 8). While these associations may partially reflect causal interactions between microbes, the full landscape of interdependencies is likely much richer and more nuanced in ways that we are just starting to grasp (9–13). Indeed, interactions may be defined and measured in many different ways (14, 15), for example, by evaluating direct contact between cells (16, 17), physical proximity (18, 19), the cost of producing exchanged metabolites (20–22), and the type of chemical mediators involved (23, 24). These factors are crucial in determining the emergence and consequences of an interaction beyond its ecological classification (mutualism, competition, etc.), and can provide a more complete view of microbial ecosystem properties, which is helpful for building mathematical models of community dynamics.

Advances in metabolomics, transcriptomics, and high-throughput culturing platforms are beginning to produce a growing body of data on the mechanisms and environmental dependencies exhibited by microbial interactions (25–28). While this wealth of information has the potential to enhance our knowledge of specific microbial interrelationships, it poses the new challenge of finding an appropriate framework to describe interactions in a way that efficiently encompasses their diversity and complexity (Fig. 1A) (29–31).

**FIG 1:**
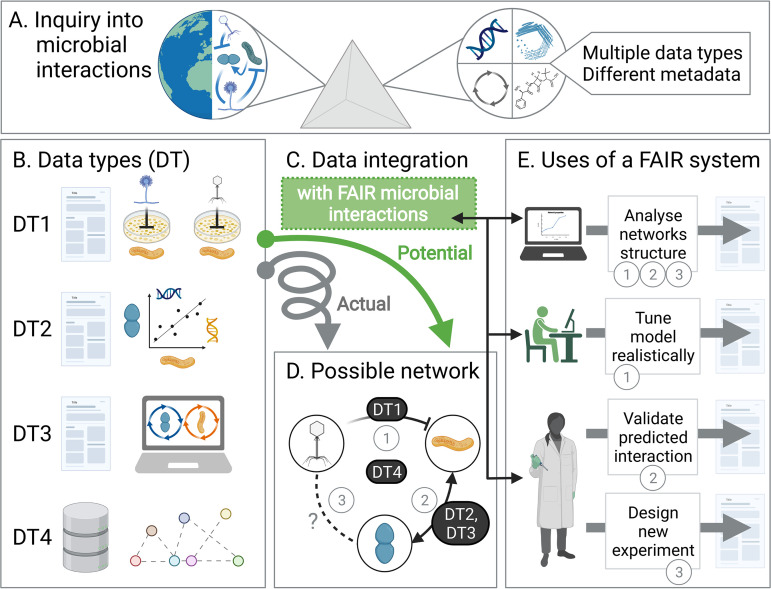
Applying a FAIR system to the study of microbial interactions and correlations. (A–B) Studies that investigate microbial interactions gain relevant insights through the generation of multiple types of data and metadata (DTs). As representative examples of microbe–microbe interactions, we may consider fungi–bacteria or phage–bacteria cultivation experiments (DT1), correlations based on amplicon sequence or operational taxonomic unit counts (DT2), and flux balance models of genome-scale metabolic networks of two or more species (DT3). While specific databases (DT4 and Table 1) that compile these data sources exist, they lack a common reporting standard, which hinders downstream application and integration. (C) We envision a systematic approach for reporting microbial interactions following the principles of Findability, Accessibility, Interoperability, and Reusability (FAIR). (D) A FAIR representation of microbial interaction data, based on common identifiers for microorganisms and specific encodings of interactions and uncertainty, can enable new insights that bridge subdisciplines and generate predictions of new interaction networks. (E) Scientists spanning diverse areas of microbiome science can benefit from FAIR reporting of interactions. For example, a network scientist could identify common structures relying on a broader corpus of interactions, a modeler could more easily identify specific interactions to realistically simulate ecological dynamics, and an experimentalist could assess whether a novel interaction has been reported in other hosts or contexts. All these uses of the framework would lead not only to new scientific insights and more streamlined contribution to data collections, but also to growing interconnectedness within the diverse field of microbiome science.

Addressing this challenge would allow us to dynamically and continuously combine diverse sources of data (Fig. 1B and C) to yield insights that could not be obtainable from individual data sets. For example, one could use an open interaction database to easily fetch experimentally-grounded parameters (e.g., metabolite uptake/secretion rates) for simulating dynamics of microbial food webs and to predict the general conditions that determine the stability of a community (Fig. 1D) (32). As another example, network scientists and ecologists could use the same database to assess how widespread particular interactions are (33), or to identify network structures that are common across biomes or taxonomic groups, refining our understanding of how microbial ecosystems assemble (Fig. 1E) (34, 35). These inferences could then be used to clarify the connection between causal interactions and observed co-occurrence patterns—distinct data types whose combination can advance the understanding of how different microbial relationships affect community function, dynamics, and resilience. Finally, simultaneous mining of multiple data sets would enable searches for examples of specific interactions (Fig. 1E), to (i) identify interactions that occur robustly irrespective of biome and experimental details, (ii) facilitate the bottom-up design of synthetic consortia by complementing existing approaches (36–38), or (iii) help create experimentally-verified data sets for benchmarking microbial inference methods (39).

More generally, the prevalence and effects of specific interaction attributes (e.g., dependence on specific resources (40), strain-level physiological differences (41), and definitions of ecological outcomes (8, 42)) across organisms and ecosystems could be quantified and compared, contributing to an enhanced understanding of the general ecological principles that govern living systems.

Despite this promising prospect, several factors complicate the integration of microbial interaction data. Among these, of particular importance is the fact that the majority of interaction data are not accessible outside the original study in which they were reported and often appear in the form of arbitrarily formatted tables. For this reason, efforts have been made to create interaction databases with standardized formatting (Table 1) (43–47). While these represent useful resources for finding specific interacting participants from diverse microbiomes, such databases are often limited to one or a few types of data. Nonetheless, these efforts follow in the rich history of endeavors within biology aimed at standardizing and sharing data and computational models of biological systems (47–51). For example, one of the most prominent early cases for the need for standardization was the explosion of high-throughput data generated from DNA microarrays at the end of the 1990s. It is particularly telling that a commentary article accompanying the paper that proposed MIAME (Minimum Information About a Microarray Experiment) (50) was titled “Microarray Standards at Last” (52), capturing the acknowledged need for appropriate reporting standards at the time. As suggested by the title, MIAME was not created overnight, but rather entailed a careful process that integrated viewpoints from multiple stakeholders to create a useful and accepted reporting framework that enhanced the reproducibility of results and the drawing of broader insights from integrated sources of data. To begin a similar journey, we propose that a greater focus on reporting interaction attributes and mechanisms using standardized formats will open up important opportunities for the microbiome field, and outline specific steps that can be taken to reach this potential.

**TABLE 1:** Databases containing microbial interactions and their relevant FAIR features^*a*^

Database	Website	Reference	Relevant features
Microbial Interaction Network Database (MIND)	microbialnet.org	43	Contains microbe–microbe interactions categorized by environmental contexts and metadata. Microorganisms involved are mapped to their NCBI Taxonomy ID to facilitate comparisons.
Protist Interaction DAtabase (PIDA)	github.com/ramalok/PIDA	44	Contains literature-extracted microbe–microbe interactions categorized by interaction source and type (ecological). Microorganisms are mapped using Genbank accession nos. when appropriate.
Microbiota-Active Substance Interactions database (MASI)	http://www.aiddlab.com/MASI/	45	Contains microbe–drug and microbe–disease interactions with particular emphasis on linking to external resources. Microorganisms are mapped using NCBI Taxonomy ID, and molecules are mapped using PubChem ID.
The Bacterial Diversity Metadatabase (BacDive)	bacdive.com	57	Contains integrated bacterial information on culture conditions and physiology that are accessible interactively and programmatically. Deposited strains are mapped to their NCBI Taxonomy ID and culture collections persistent identifiers.
Web of Microbes (WoM)	webofmicrobes.org	46	Contains microbe–metabolite–microbe interactions for multiple media conditions. Exptl information linking metabolite (environment) and microbe compatibility is displayed in a browsable and searchable manner.
Global Biotic Interactions database (GloBI)	globalbioticinteractions.org	47	Contains descriptions of relationships between biological entities in general (i.e., species–species, species–habitat, etc.). Entities are indexed based on independent repositories that comply with light formatting and follow a strict ecology-based interaction ontology.

a In addition to databases describing intermicrobial interactions, this table also contains databases that contain frameworks to compile a variety of biological relationships (e.g., GloBI), as well as microbial databases aggregating scattered knowledge and promoting data sharing (e.g., MASI and BacDive).

## A FAIR REPRESENTATION OF MICROBIAL INTERACTIONS

We specifically envision the adoption of data sharing and stewardship practices that would enable microbial interaction data to fulfill the principles of Findability, Accessibility, Interoperability, and Reusability (FAIR). These principles, first formally presented in 2016 to address growing challenges in scientific data management, serve to guide efforts that aim to improve access to reliable and reproducible scholarly data (53) and have already been adopted as an important component of microbiome data management (1, 6, 54–58). We therefore focus our present discussion on two concrete efforts that can be initiated in order to make data on intermicrobial interactions more FAIR, namely (i) the creation and/or adoption of open web infrastructures for cataloging and making data from disparate sources available to the community (hence Findable and Accessible), and (ii) the adoption of a minimal set of metadata requirements that are human- and machine-readable (i.e., Interoperable and Reusable).

### 1: An open web catalog for findable and accessible microbial interaction data.

It is difficult to imagine how to make progress on Findability and Accessibility without a centralized resource that is capable of capturing the wide breadth of interactions currently available only in individual publications or split into type-specific repositories (Fig. 1B and Table 1), and designed to be able to grow to accommodate newly generated data. Such a resource could be generated via multiple strategies, including the following:

- **Integrating existing repositories of microbial interaction data into an established infrastructure.** Several existing infrastructures could in principle serve this purpose. To illustrate the potential advantages and challenges of this strategy, we may consider as an example Global Biotic Interactions (GloBI) (47), a metadatabase for sharing and analyzing species interaction data. It provides a searchable platform to identify specific interactions based on the organisms involved and the relationships they experience (e.g., X preys on, hosts, is symbiont of Y), and can therefore serve as a structure for integrating a wider breadth of microbial interaction data and their attributes. Nonetheless, as GloBI considers the species level as the most phylogenetically precise, it may not easily capture strain- or mutant-specific interactions common in microbial ecology research (59). Integration into GloBI would also require amending existing metadata items not applicable to microbes, as well as the ontologies of interactions (apart from “interacts with,” “parasitizes,” “ecologically co-occurs with”) to match features of microbial interactions.
- **Using existing database-building tools and available metadata.** For example, one could consider using the recently-published tool mako (35) to create a database by importing network files or deposited sequences to be analyzed. If these input files contain metadata on nodes, edges, or samples from which sequences were obtained, the metadata in question can be readily propagated into the database. However, mako is currently limited to undirected interactions and to creating a local interaction database, which complicates continuous online access and editing.
- **Establishing a new database specific to microbial interactions.** Such a database could be designed from the ground up to more flexibly store multiple types of microbial interactions (e.g., co-occurrences, causal interactions, and higher-order interactions), as well as their attributes. This approach could apply several modalities for importing and organizing data in a standardized or automated way, facilitating the incorporation of data from individual studies as they are published. Recent manually-compiled catalogs of interactions and their attributes (14, 15) as well as of tools to convert them into searchable online resources (cpauvert.github.io/mi-atlas) may serve as small-scale examples for planning such a larger-scale resource.

### 2: A minimal set of metadata requirements for interoperability and reusability.

While a centralized database would lay a foundation for FAIR microbial interaction data, its impact would remain limited if its contents cannot be easily updated by scientists and accessed by humans and machines. We therefore also advocate for the inclusion of metadata with reports of interactions as a way to promote interoperability and reusability. While convergence to specific guidelines will require significant community discussions and buy-in from stakeholders, we propose that the following four categories of metadata could serve as a starting point: microbial entities, interaction inference methods, interaction context, and attributes. These are described in detail in Table 2 and are outlined as follows:

**TABLE 2:** List of proposed metadata for minimum information about intermicrobial interaction data^*a*^

Metadata	Level	Description
A. Which microbial entities are involved?		
participants	M	Comma-separated list of the microbial entities’ names, with descriptions of any genetic manipulations performed.
tax_id	M	Comma-separated list of the matching identifiers from the NCBI Taxonomy at the relevant taxonomic level. (e.g., NCBI:txid1043002, NCBI:txid411903). Novel taxa lacking identifiers are denoted by N/A^*b*^.
sequence_id	R	Comma-separated list of the accessions to the matching sequence data (e.g., genome, marker gene sequence). Taxa from presequencing era articles could be denoted by N/A.
env_origin	X	Term from the Environmental Ontology indicating from which biome the microbial entities originate (e.g., soil [ENVO:00001998]).
source_collection	X	Comma-separated list of the source of the participants engaging in this interaction: isolation, commercial collection, academic collection
B. How was the interaction uncovered?		
evidence_type	M	Type of evidence used to determine the interaction using the Evidence and Conclusion Ontology. At least one of the following broader terms are required: exptl [ECO:0000006], computational [ECO:0007672], or both [ECO:0007661].
method_type	R	One or several of the following types of methods used to determine the interaction:
		• Simulation-based (e.g., generalized Lotka-Volterra model, genome-scale metabolic model)
		• Microscopy-based (e.g., co-localization with fluorescent markers, assisted motility)
		• Cultivation-based (e.g., continuous co-culture in bioreactor, co-plating on solid media)
		• Sample-based (e.g., co-occurrences drawn from analyses of abundances obtained from *in situ* or *in vivo* sampling).
reference	M	Persistent identifier (e.g., DOI or URL) to a resource, script, or article, documenting the method.
software_parameters	X	Name, version, and parameters of the software used, using the following syntax: {software}:{version}:{parameters}.
C. What is the environmental context of the experiment?		
env_broad_scale	R	Biome term from the Environmental Ontology. Engineered ecosystems such as bioreactors, agar plates, or computational models use N/A.
Site	X	Cellular component (from the Gene Ontology) involved in the interaction: cytoplasm [GO:0005737], membrane [GO:0016020], or the extracellular region [GO:0005576].
compounds	X	One or several chemical entities involved in the interaction using either broad or precise terms from the CheBI ontology with their identifiers. Example: short-chain fatty acid [CHEBI:26666], bacterial metabolite [CHEBI:76969], or penicillin [CHEBI:17334].
medium_name	X	Name of the (*in vitro* or *in silico*) cultivation medium used, with URL to the composition. Example: BHI [https://bacmedia.dsmz.de/medium/215].
rel_to_oxygen	X	Term indicating the oxygen status of the environment using terms from the MIxS: aerobic or anaerobic.
ph	X	Measurement of the pH in the environment.
temp	X	Measurement of the temp of the environment (in °C).
carbon_source	X	Term from the ChEBI indicating the specific carbon source(s) used.
nitrogen_source	X	Term from the ChEBI indicating the specific nitrogen source(s) used.
inoculation_densities	X	Comma-separated list of densities for cultivation experiments measured with the associated units in brackets: with optical density [OD 600], with colony forming units [CFU/mL].
D. What are the attributes of the interaction?		
participant_outcomes	R	Comma-separated list of the outcome for each participant: 0 (not affected), 1 (positively affected), –1 (negatively affected), N/A.
ecological_outcome	X	For pairwise interactions, one or several terms describing the overall outcome (https://doi.org/10.2307/1307540): co-occurrence, cooperation, commensalism, exploitation, amensalism, competition, neutralism.
strength	X	Numerical value quantifying the intensity of the interaction. Example: the inhibition score after co-plating, the correlation value between relative abundances, or estimate of generalized Lotka-Volterra model parameters.
dependencies	X	One or several of the following terms: contact, time, space.
keywords	X	Comma-separated list of terms providing more detail on the broader context of the interaction (e.g., disease-related, biofuel production, uncultivable organisms, metabolic engineering).
	
notes	X	Open text field for additional relevant comments.

a The level of requirement of the metadata is either mandatory (M), recommended (R), or optional (X). Four general questions regarding interactions divide the metadata into four categories (A–D).

b NA, not applicable.

- **Microbial entities.** The species (and strain, if relevant) names of each of the microbes participating in an interaction should be provided, e.g., in a comma-separated list, along with their taxonomic accession numbers and eventually their sequence identifier (Table 2A). These lists would also accommodate interactions that cannot be easily described via a pairwise representation. Interaction attributes and effects specific to each participant could be matched with each identifier.
- **Interaction inference methods.** Despite being challenging to standardize, documentation of the methods that were used to identify an interaction represent highly relevant metadata. As a first step, the evidence for the interaction in question can be broadly categorized using the Evidence and Conclusion Ontology (60), which would indicate whether experimental or computational methods (or both) were used. We also propose a more specific metadata item for the type of computational or experimental method used (e.g., simulation, microscopy, cultivation, and sampling) (Table 2B). Lastly, the relevant publication, code, detailed protocols, and other literature-based evidence should be accessible via persistent identifiers (e.g., DOIs).
- **Interaction context.** The environmental context of the interaction—such as the biome (e.g., host-associated, synthetic)—could be documented using the Environment Ontology (61) or propagated from the samples used to infer the interaction. Cultivation conditions could also be integrated following the standards established by databases of bacterial isolates (57) and extended to co-cultures. Relevant metadata are proposed in Table 2C with an emphasis on linking values to existing resources such as the Gene Ontology for cellular components, the Chemical Entities of Biological Interest Ontology for compounds, and the Genome Standards Consortium (51) for the oxygen status of the environment.
- **Interaction attributes.** Defining an interaction’s type (e.g., cooperation, antagonism, association, pairwise or higher-order, etc.) is also not trivial, but could be guided by incorporating existing ontologies such as the active list maintained by the OBO Foundry (62). Several other frameworks exist to describe interaction types such as GloBI, Population and Community Ontology (63), and Interaction Network Ontology (64). It nonetheless remains to be seen whether these ontologies are appropriate for describing all known attributes of microbial interactions, or if a larger set defined by the community is needed. In the meantime, we propose the inclusion of the ecological effect experienced by each participant or by each set of participants (positive/negative/neutral) (42), as well as of information on whether the association described is a co-occurrence by providing the associated metric strength. Lastly, we propose the inclusion of any known interaction dependencies (e.g., on spatial structure or physical contact) and any additional user-defined keywords that provide further relevant information not captured in the previous items (Table 2D).

As an example of how these metadata can be compiled for different data types (Fig. 1B), we have used them to describe three interactions gathered from the literature (Table 3).

**TABLE 3:** Three microbial interaction data types represented according to the proposed metadata guidelines in Table 2^*a*^

Metadata	Example A	Example B	Example C
Microbial entities			
participants	Azotobacter vinelandii DSM 85, Chlamydomonas reinhardtii strain 187, *Alternaria* sp. GYI-051221	Acidobacteria, Gammaproteobacteria	Bacteroides caccae ATCC 43185, Lactobacillus rhamnosus GG
	NCBI:txid354, NCBI:txid3055, NCBI:txid667197	NCBI:txid57723, NCBI:txid1236	NCBI:txid411901, NCBI:txid568703
sequence_id	N/A, N/A, FJ627005.1		NZ_CP022412.2, NZ_CP031290.1
env_origin		soil environment [ENVO:01001044], soil environment [ENVO:01001044]	digestive tract environment [ENVO:01001033], digestive tract environment [ENVO:01001033]
source_collection	commercial collection, commercial collection, isolation		
Interaction inference methods			
evidence_type	experimental evidence used in manual assertion [ECO:0000269]	high throughput evidence used in automatic assertion [ECO:0006057]	computational evidence [ECO:0007672]
method_type	cultivation-based, microscopy-based	sample-based	simulation-based
reference	https://doi.org/10.1007/s12223-010-0067-9	https://doi.org/10.1038/ismej.2011.119	https://doi.org/10.1038/nbt.3703
Interaction context			
env_broad_scale		soil environment [ENVO:01001044]	
compounds	chlorophyll [CHEBI:28966], cystathionine [CHEBI:17755]		alanine [CHEBI:16449], asparagine [CHEBI:22653], nicotinic acid [CHEBI:15940], lactate [CHEBI:24996]
medium_name	Azotobacter Medium [https://bacmedia.dsmz.de/medium/3]		DMEM 6429 (+ vitamin K, hemin, and arabinogalactan)
rel_to_oxygen	aerobic		anaerobic
temp	25		37
Interaction attributes			
participants_outcome	1,1,1	N/A^*b*^	1,1
ecological_outcome	cooperation	co-occurrence	
strength		0.66	
dependencies	contact		

a Included here are an interaction from an alga-bacteria- fungi cultivation experiment (A), a co-occurrence analysis of bacterial operational taxonomic units (B), and a genome-scale metabolic model (C).

b NA, not applicable.

## OUTLOOK

The practices, standards, and use cases we have outlined here are by no means exhaustive, but are rather meant to catalyze further discussion on ways to improve the access to and usability of data on microbial interactions and their attributes. We believe the time is opportune for such discussions to take place, not only due to the rapidly growing body of data on microbial interactions and their mechanisms, but also because of a growing momentum within the microbiome community to improve the reliability and reproducibility of research outputs. These are exemplified by government-funded initiatives such as the National Microbiome Data Collaborative (NMDC, USA (56, 65) and the National Research Data Infrastructure (NFDI4Microbiota, Germany; (https://nfdi4microbiota.de), which advocate for the adoption of reporting standards for microbiome data. As with existing accepted data reporting standards, any proposed global framework for describing microbial interactions must be shaped by its various stakeholders, including computational and empirical researchers, industry representatives, funding agencies, educational users, and publishers. Such involvement would enable any formalism to be flexible and broadly embraced, as opposed to a rigid standard with little endorsement or room for growth.

Bearing these considerations in mind, we suggest the following roadmap toward FAIR microbial interaction data. First, we call for increased discussions within the scientific community to select and prioritize the interaction features that are most useful to report. These can be carried out via dedicated workshops that, in addition to biologists, could include philosophers of biology interested in microorganisms, as well as physicists and mathematicians who can help define the qualitative and quantitative nature of intermicrobial interactions and their important attributes. This first community-driven effort could lead to the creation of a reporting standard, extending our suggestions in Table 2 to a more mature “Minimal Information for Intermicrobial Interactions” definition similar to those for publishing microarray data (50) and genome sequences (51, 66, 67), or for assessing the quality of genome-scale metabolic models (68). Second, these metadata suggestions could be further implemented as usable formats such as SBML (49, 69) or BIOM (70), which enable the standardized export and sharing of genome-scale models and count data, respectively. As such, data scientists and bioinformaticians could take part in hackathons to develop such a toolbox with standardized file formats, converter scripts, and validators to streamline the adoption of microbial interaction metadata. Third, we envision teams of investigators and students gathering for “annota-thons” to collaboratively extract knowledge from the microbial interaction literature and use the aforementioned toolboxes to compile the relevant metadata, ensuring that an open web catalog of microbial interactions truly relies on known published material. Last, the rise of an open community, willing to quickly share protocols and methods of scientific projects enabled by FAIR microbial interaction data resources, would provide further incentives for adoption of standard formats, creating a positive feedback loop that could accelerate benefits for the whole community and pave the way for major integrative and collaborative advances in microbiome research.
